# Modelling the cost-effectiveness of brief aftercare interventions following hospital-treated self-harm

**DOI:** 10.1192/bjo.2023.525

**Published:** 2023-08-01

**Authors:** Long Khanh-Dao Le, Anna Flego, Karolina Krysinska, Karl Andriessen, Piumee Bandara, Andrew Page, Marisa Schlichthorst, Jane Pirkis, Cathrine Mihalopoulos, Greg Carter, Lennart Reifels

**Affiliations:** Health Economics Division, School of Public Health and Preventive Medicine, Monash University, Melbourne, Victoria, Australia; Centre for Mental Health, Melbourne School of Population and Global Health, The University of Melbourne, Victoria, Australia; Translational Health Research Institute, Western Sydney University, New South Wales, Australia; School of Medicine and Public Health, University of Newcastle, New South Wales, Australia

**Keywords:** Brief aftercare intervention, cost-effectiveness, self-harm, suicide, suicide attempt

## Abstract

**Background:**

Prior self-harm represents the most significant risk factor for future self-harm or suicide.

**Aim:**

To evaluate the cost-effectiveness of a theoretical brief aftercare intervention (involving brief follow-up contact, care coordination and safety planning), following a hospital-treated self-harm episode, for reducing repeated self-harm within the Australian context.

**Method:**

We employed economic modelling techniques to undertake: (a) a return-on-investment analysis, which compared the cost-savings generated by the intervention with the overall cost of implementing the intervention; and (b) a cost–utility analysis, which compared the net costs of the intervention with health outcomes measured in quality-adjusted life years (QALYs). We considered cost offsets associated with hospital admission for self-harm and the cost of suicide over a period of 10 years in the base case analysis. Uncertainty and one-way sensitivity analyses were also conducted.

**Results:**

The brief aftercare intervention resulted in net cost-savings of AUD$7.5 M (95% uncertainty interval: −56.2 M to 15.1 M) and was associated with a gain of 222 (95% uncertainty interval: 45 to 563) QALYs over a 10-year period. The estimated return-on-investment ratio for the intervention's modelled cost in relation to cost-savings was 1.58 (95% uncertainty interval: −0.17 to 5.33). Eighty-seven per cent of uncertainty iterations showed that the intervention could be considered cost-effective, either through cost-savings or with an acceptable cost-effectiveness ratio of 50 000 per QALY gained. The results remained robust across sensitivity analyses.

**Conclusions:**

A theoretical brief aftercare intervention is highly likely to be cost-effective for preventing suicide and self-harm among individuals with a history of self-harm.

Every year, over 700 000 individuals tragically lose their lives to suicide, representing a global age-standardised suicide rate of 10.5 per 100 000 people in 2016.^1^ In 2020, Australia had a suicide death rate of 12.1 per 100 000 people, along with a self-harm hospital admission rate of 113 per 100 000 people.^2^ Current estimates of hospital-treated self-harm based on institutional administrative data are likely to underestimate the overall burden of self-harm in the population. This is because many instances of self-harm do not lead to hospital admission. It is important to note that prior self-harm represents the most significant risk factor for future self-harm or suicide.^3,4^ For example, Olfson et al^3^ found that the rate of repeated self-harm during the 12 months after non-fatal self-harm was 37 times higher than in the matched general population. Furthermore, self-harm has a significant impact on health outcomes and productivity loss.^5,6^ Nguyen et al^7^ found that only one-third of people who had self-harmed and were discharged from hospital achieved ‘good recovery’ (as measured by the Glasgow Outcome Scale Extended) and, importantly, only half returned to work within 24 months of their self-harm. For these reasons, it is important to provide effective and cost-effective care for those who self-harm.

The body of evidence supporting the effectiveness of aftercare interventions aimed at preventing recurring self-harm episodes is steadily growing. Aftercare interventions are defined as interventions providing care for someone after they have had a self-harm episode. Research has demonstrated that psychological or psychosocial aftercare interventions are linked to a reduced risk of subsequent self-harm.^8^ However, it is worth noting that these interventions can be resource-intensive, as they often require specialised clinician training, which may not always be feasible to implement.^9^ Furthermore, those who self-harm have demonstrated low adherence to intensive psychological and psychosocial interventions.^10,11^ In view of this, brief aftercare interventions that focus on preventing recurrent self-harm and involve direct ongoing contact, along with the option for re-contact with clinical services if needed, may serve as a crucial and viable alternative.^12^ There are several forms of brief aftercare interventions, including supportive phone calls (i.e. telephone outreach), with or without care coordination, as well as follow-up services provided after the patient has been discharged from the acute care unit or emergency department.^13^ A recent meta-analysis indicated a noteworthy decrease in the occurrence of subsequent self-harm episodes per individual when employing brief aftercare interventions that incorporated at least one of four main components: brief contact interventions, care coordination, safety planning interventions and/or other brief therapies. This reduction was observed when comparing these interventions with control groups that did not receive any intervention (odds ratio (OR): 0.69; 95% CI: 0.53 to 0.89).^13^

There is a relative paucity of economic evaluations of brief aftercare interventions designed to prevent or reduce subsequent self-harm episodes, although there have been some economic studies of brief aftercare interventions (i.e. interventions with one or two of the components identified by Doupnik et al^13^). In particular, a pre–post study conducted in the USA found that for every US$1 invested in delivering follow-up calls after discharge to individuals who had been admitted to hospital or presented to an emergency department following self-harm, the estimated return on investment (ROI) ranged from US$1.7 to US$2.43, depending on whether individuals had commercial insurance or were covered by Medicaid.^14^ A recent model-based economic evaluation indicated that telephone outreach was more costly but more effective than usual care (i.e. defined as only one-third of people receiving an average of one initial diagnostic evaluation plus two 45-min psychotherapy sessions during the 12 weeks post-discharge), with an incremental cost-effectiveness ratio (ICER) of US$4300 per life year under a health sector perspective.^15^ The combination of universal screening in the emergency department and a telephone-based intervention provided over a period of 12 months after the emergency department visit was deemed cost-effective, with an ICER of US$5020 per additional attempt or death prevented.^16^ It is noteworthy that in the existing economic evidence, the effectiveness of brief aftercare interventions was estimated from a single study rather than a meta-analysis of individual studies; moreover, the single study was conducted in a USA context, and so its findings may not be applicable to other settings.

The current study is the first to evaluate the cost-effectiveness of a theoretical brief aftercare intervention that included all of the components identified by Doupnik et al^13^ (i.e. brief contact interventions, safety planning interventions, care coordination and/or other brief therapies) within the Australian context. We used different economic frameworks (ROI and cost–utility analysis; CUA) in our study and examined whether the brief aftercare intervention delivered to those who had been admitted to a hospital unit or presented to an emergency department for self-harm provides value for money.

## Method

The current study involved an economic evaluation of a theoretical brief aftercare intervention over a period of 10 years. The study followed the guidelines and checklist provided by the Consolidated Health Economic Evaluation Reporting Standards to ensure comprehensive and standardised reporting of the health economic evaluation, as outlined in Supplementary Appendix 1 available at https://doi.org/10.1192/bjo.2023.525. The approach to modelling intervention effectiveness was based on a prior model developed to evaluate the cost-effectiveness of four suicide prevention strategies that formed part of the ACE Prevention study.^17^ The effectiveness of a theoretical brief aftercare intervention was based on an effect size derived from a recent meta-analysis by Doupnik et al,^13^ whereas the direct intervention cost was based on estimates from the Australian brief aftercare programme for prevention of repeated self-harm.^18^ In this study, a partial societal perspective was used to assess both the costs and health benefits. That meant that healthcare costs related to hospital admissions due to self-harm and the healthcare cost offsets and cost of productivity loss related to suicides were included in the base case analysis. The analysis did not consider time or travel costs incurred by people who had self-harmed and received brief aftercare intervention or by their carers and/or family members.

This study used two different economic evaluation frameworks: (a) an ROI analysis, which compared the cost-savings generated by the intervention with the total cost of implementing the intervention; and (b) a CUA, which compared the net costs of the intervention to the net health outcomes measured in quality-adjusted life years (QALYs). The results of the study are presented in the form of ROI ratios (cost-saving divided by intervention cost) and ICERs, representing the cost per QALY gained. The ICERs were evaluated using the commonly accepted willingness-to-pay threshold of $50 000 per QALY. The study used a 10-year time horizon and adjusted all costs to 2018 Australian dollars (AUD) using the most recent relevant health price deflators. Both costs and health benefits were discounted at a rate of 3% *per annum*.

### Intervention description

As identified in the meta-analysis by Doupnik et al,^13^ various components can be included in brief aftercare interventions. For the purpose of costing a pathway of a theoretical brief aftercare intervention in the Australian context, we chose an implemented intervention, the Way Back Support Service, which includes four components of a brief aftercare intervention.^19^ The Way Back was a non-clinical service for people aged 15 or over who presented to the emergency department or required hospital admission following self-harm and had the aim of connecting them to care.^19,20^ The Way Back included (a) follow-up telephone contacts, (b) care coordination, (c) safety planning and (d) brief therapeutic intervention. A support coordinator initiated contact with the person after receiving their referral from the hospital to explain the scope and role of the support offered by the Way Back. An initial assessment appointment was organised post-discharge to identify the goals and needs of the consumers, to develop a safety plan and assist people to stay connected to informal and formal supports tailored to their individual needs and preferences.^19^ In this economic evaluation, the intervention was modelled for the entire population of people with self-harm in Australia, with the assumption that they would all have access to the Way Back.

### Intervention effectiveness

We based our estimates of the effectiveness of the intervention on the recent meta-analysis of brief aftercare contact interventions by Doupnik et al,^13^ in which a pooled effect size was calculated based on seven randomised controlled trials of brief aftercare suicide prevention interventions. The pooled estimate indicated significantly lower odds of subsequent self-harm reattempts (pooled OR: 0.69; 95% CI: 0.55 to 0.87) following an intervention compared with no intervention. There was no evidence pertaining to whether this reduction was maintained over time after the first year. Therefore, in the base case analysis, our model assumed a 50% decay effect regarding intervention effectiveness over the first 5 years and no effect of the intervention after that. Similar assumptions have been made in previous Australian economic evaluations of preventive interventions for mental disorders and suicide. We also conducted a sensitivity analysis, which assumed conservatively that the intervention had an effect only up to 12 months (and a 100% decay effect thereafter). Furthermore, it is noteworthy that the effectiveness of the Way Back used for intervention costing was not captured in this meta-analysis,^13^ given that it had been recently published and did not meet the study design criteria to be included in the meta-analysis (i.e. it was a historical control study rather than a randomised controlled trial).^18^

### Comparator (or no investment scenarios)

A ‘do nothing’ comparator or no intervention was chosen because it is not clear what constitutes standard or routine practice for people presenting with self-harm in the Australian context. This also meant that people could receive treatment as usual regardless of whether they received the brief aftercare intervention. The cost of a comparator, thus, was not included in the analysis.

### Eligible population

The eligible population for the modelling was all individuals aged 15 years or older who presented to hospital following an episode of self-harm (similar to the eligibility criteria for the Way Back).^19^ According to a process and impact evaluation of the Way Back, of 1,120 participants initially identified, 821 people (approximately 73.3%) were eligible to receive the service. Therefore, approximately 73.3% were assumed to have taken up Way Back services in this study.^20^

### Health benefit modelling

**Fig. 1:**
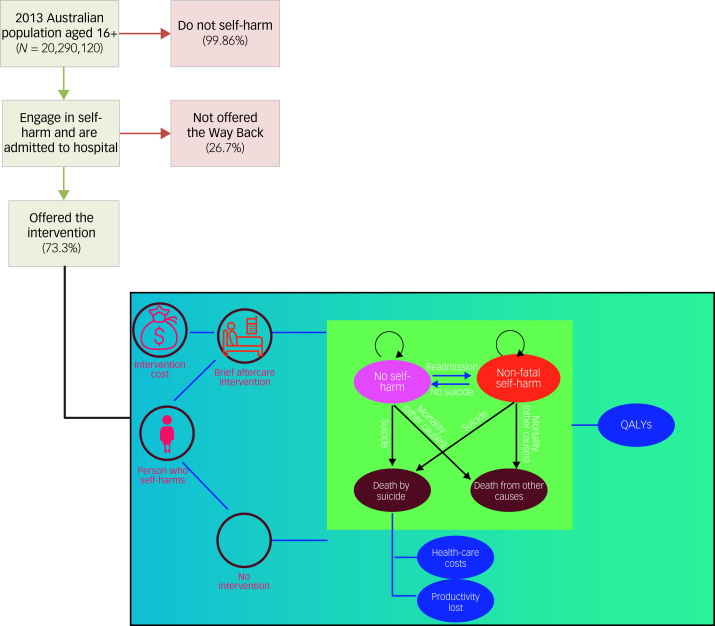
The Way Back Service pathway and schematic model. QALYs, quality-adjusted life years.

We adopted a simple population-level Markov model developed by Mihalopoulos et al^21^ which simulated the transitions of each age–sex cohort among three different health states – no self-harm, self-harm and deceased (by suicide or other causes) – over a period of 10 years. The selection of a 10-year time horizon was based on previous evidence showing that it provides an adequate duration to evaluate the beneficial impacts of preventive interventions.^22^ People who engage in self-harm and are admitted to hospital enter the model and either take up or do not take up the brief aftercare intervention. At the end of the cycle, everyone enters the Markov phase of the model where they: (a) do not repeat self-harming behaviour; (b) repeat self-harming behaviour (with a non-fatal outcome); (c) die by suicide; or (d) die from other causes. The state transition diagram is presented in Fig. 1. The number of people who had a self-harm episode was determined from the rates of self-harm hospital admissions in Australia.^2^ The probability of fatal and non-fatal repeated self-harm each year following the index self-harm episode was estimated from the most recently published relevant international review of cohort studies and randomised controlled trials.^23^ Reduced probability of self-harm was included in the model by applying the odd ratios (converted to a probability) from Carroll et al^23^ to the naturalistic estimates of self-harm described by Carrol et al.^23^ The model quantified the impact on health of a lower probability of repeated self-harm and subsequent suicide in the intervention scenario versus no intervention.

### Cost-effectiveness frameworks

The ROI framework was chosen for the primary analysis comparing the cost-savings produced by the intervention relative to costs of the intervention. This ratio is technically a benefit/cost ratio, which has been used in previous ROI studies in Australia^24^ and in the UK.^25^ Interventions with ROI ratios greater than one are considered cost-saving, indicating that the cost-savings outweigh the total cost of the intervention. A CUA was adopted for the secondary analysis, in which QALYs were used as a metric to measure health gains. QALYs combine both the quantity gains from reduced mortality and the quality gains from improvements in health-related quality of life. Utility weights were employed to adjust each year lived in a specific health state on a scale ranging from 0 (representing death) to 1 (indicating perfect health). The utility weights of health states for those who do not engage in self-harm or engage in self-harm that is not fatal were estimated to be 0.64 (95% CI: 0.33 to 0.95) and 0.54 (95% CI: 0.29 to 0.79), respectively, as sourced from a population study.^26^ Among those who do not engage in self-harm, the utility weights of some individuals’ health states may be low (i.e. 0.64), indicating the impact of previous experience of self-harm. Given the overlap in 95% confidence intervals of utility scores between these health states, in the probabilistic analysis, the utility score of the self-harm health state was constrained to the utility score of the non-fatal self-harm health state plus the difference in utility scores between these health states (i.e. 0.1). The cost-effectiveness results were presented in the form of an ICER, which was calculated by dividing the difference in costs between the intervention and no intervention by the difference in QALYs gained. Table 1 provides a comprehensive overview of all input parameters and their corresponding uncertainty ranges related to health benefits in this study.

**Table 1:** Input parameters and uncertainty ranges for health benefit and costing

Parameter	Value and uncertainty range	Uncertainty distribution	Source(s)
Risk ratio for the a brief aftercare intervention (by follow-up period)	6-month follow up: 0.54 (95% CI: 0.34 to 0.85) 12-month follow up: 0.80 (95% CI: 0.65 to 0.98)	Log-normal	A systematic review and meta-analysis^13^
2018 Australian population	15–24 years: M 1 655 870; F 1 575 033 25–34 years: M 1 862 605; F 1 877 533 35–44 years: M 1 651 132; F 1 665 139 45–54 years: M 1 567 888; F 1 631 171 55–64 years: M 1 411 373; F 1 477 355 65–74 years: M 1 090 144; F 1 135 021 75–84 years: M 552 254; F 633 917 85+years: M 191 285; F 312 400 Total: M 9 982 551; F 10 307 569	Fixed	ABS estimated resident population^27^
Intentional self-harm rates (hospital admissions)	15–19 years: M 0.17%; F 0.60% 20–24 years: M 0.16%; F 0.33% 25–29 years: M 0.14%; F 0.21% 30–34 years: M 0.12%; F 0.17% 35–39 years: M 0.13%; F 0.17% 40–44 years: M 0.12%; F 0.17% 45–49 years: M 0.12%; F 0.17% 50–54 years: M 0.09%; F 0.15% 55–59 years: M 0.08%; F 0.09% 60–64 years: M 0.05%; F 0.07% 65–69 years: M 0.04%; F 0.05% 70–74 years: M 0.03%; F 0.04% 75–79 years: M 0.03%; F 0.04% 80–84 years: M 0.04%; F 0.04% 85+ years: M 0.06%; F 0.03%	Fixed	AIHW suicide and self-harm monitoring – National Hospital Morbidity Database^2^
Incidence of non-fatal repeated self-harm	1 year: 16.3% (95% CI: 15.1 to 17.7) 2 years: 16.8% (95% CI: 14.7 to 19.2) 5 years: 22.4% (95% CI: 17.0 to 28.9)	Beta	Carroll et al^23^
Incidence of fatal repeated self-harm	1 year M: 2.7% (95% CI: 1.8 to 4.0) F: 1.2% (95% CI: 0.7 to 1.9) 2 years: 2.1% (95% CI: 1.6 to 2.8) 5 years: 3.9% (95% CI: 3.2 to 4.8) 10 years: 4.2% (95% CI: 3.1 to 5.6)	Beta	Carroll et al^23^
Other-cause mortality	15–19 years: M 0.04%; F 0.02% 20–24 years: M 0.06%; F 0.02% 25–29 years: M 0.06%; F 0.02% 30–34 years: M 0.08%; F 0.03% 35–39 years: M 0.11%; F 0.06% 40–44 years: M 0.14%; F 0.09% 45–49 years: M 0.22%; F 0.13% 50–54 years: M 0.32%; F 0.20% 55–59 years: M 0.49%; F 0.30% 60–64 years: M 0.75%; F 0.45% 65–69 years: M 1.12%; F 0.67% 70–74 years: M 1.80%; F 1.13% 75–79 years: M 3.07%; F 2.04% 80–84 years: M 5.64%; F 3.88% 85+ years: M 13.6%; F 11.7%	Fixed	ABS^28^
Utility weight for non-fatal self-harm	0.54 (95% CI: 0.29 to 0.60)	Beta	van Spijker et al^26^
Utility weight for non-self-harm	0.64 (95% CI: 0.33 to 0.95)	Beta	van Spijker et al^26^
Cost of the outreach telephone intervention (adapted from the Way Back cost)	AUD$798 per person	Fixed	
Health expenditure for a suicide attempt	15–19 years: M AUD$8066; F $6320 20–24 years: M AUD$7367; F $5825 25–29 years: M AUD$8106; F $6834 30–34 years: M AUD$8752; F $8122 35–39 years: M AUD$8749; F $7718 40–44 years: M AUD$6849; F $6020 45–49 years: M AUD$7504; F $6218 50–54 years: M AUD$8352; F $7065 55–59 years: M AUD$12 248; F $9292 60–64 years: M AUD$6555; F $4780 65–69 years: M AUD$10 145; F $7520 70–74 years: M AUD$13 993; F $11 176 75–79 years: M AUD$17 249; F $14 112 80–84 years: M AUD$14 705; F $9966 85+ years: M AUD$13 774; F $15 364	Log-normal	AIHW^35^
Value of a statistical life	AUD$4.5 million	Fixed	Department of the Prime Minister and Cabinet^29^
Unit cost uncertainty	±20%	Pert	Le et al^22^
On-cost^a^	30%	Fixed	Le et al^22^

ABS, Australian Bureau of Statistics; AIHW, Australian Institute of Health and Welfare; F, female; M, male.

a. Wage rates were adjusted to incorporate 30% on-costs, i.e. additional loadings to account for administration costs, leave, superannuation, etc.

### Intervention costs

As mentioned above, the Way Back was chosen as an example for costing a theoretical brief aftercare intervention in Australia. This study assumed a steady-state scenario where the intervention operates with the presence of trained staff and the required infrastructure to deliver it effectively. Therefore, operating, and overhead costs were excluded from the analysis, although training costs during the intervention were included. The Way Back cost was assumed to be equivalent to the cost of the Way Back as implemented in the Hunter New England Mental Health Service and Hunter Primary Care in Newcastle, New South Wales, Australia. This specific site was chosen because it is the only site where a costing of the Way Back in practice has been conducted.^18^ In this costing analysis, costs of the Way Back were calculated by summing the costs for (a) staff who delivered the intervention, (b) staff training and professional development, and (c) staff travel. The total cost of the Way Back was estimated at AUD$615 per person. As the unit cost of the intervention may vary depending on the geographic area or specific context, we applied ±20% uncertainty around this unit cost.

### Cost-savings

Cost-savings included healthcare cost-savings related to averting further episodes of non-fatal self-harm and costs related to suicides. In this study, the former cost-saving was sourced from Australian Institute of Health and Welfare (AIHW) health expenditure data^2^ and the costs attributable to category U01 ‘Suicide and self-inflicted injuries’. To estimate the financial value society assigns to the reduction of death risk, one approach is to use the concept of the value of a statistical life (VSL). However, measures of QALYs and VSL may be at risk of double counting.^30^ We therefore used a weighted average of healthcare and productivity costs associated with a suicide death sourced from KPMG (2013)^31^ for estimating the cost-savings associated with suicide deaths in the base case analysis, and we used the VSL in the sensitivity analysis. The weighted average cost of a suicide reported by KPMG was AUD$714 681, estimated by summing (a) the direct costs of suicide relating to coronial injuries, police and ambulance services, and counselling support to family/friends of the person who had died, and (b) the indirect costs of suicide including the lost economic contribution of an individual owing to premature mortality.^31^

### Uncertainty and sensitivity analyses

To account for parameter uncertainty (i.e. sampling error) and its impact on the model outputs, uncertainty analyses were performed alongside each cost-effectiveness model. A Monte Carlo simulation consisting of 3000 iterations was conducted in Excel for each uncertainty analysis. This allowed the estimation of incremental costs, QALYs, ICERs and ROI ratios, along with their corresponding 95% uncertainty intervals. The uncertainty iterations were visually represented on a cost-effectiveness plane to provide a comprehensive understanding of the results. Detailed information regarding the uncertainty parameters can be found in Table 1.

To assess the robustness of the findings, sensitivity analyses were conducted, considering the impact of varying model parameters and assumptions. Five alternative scenarios were explored within these sensitivity analyses, allowing a comprehensive evaluation of the potential influence on the results.

Sensitivity analysis 1: modelling short-term intervention effectiveness only, such that the intervention was not effective after the first year (i.e. assuming a 100% decay rate after 1 year, instead of a 50% annual decay rate as in the base case analysis).

Sensitivity analysis 2: the cost associated with a death was based on the VSL at AUD$4.5 million measured in 2018 dollars recommended by the Department of the Prime Minister and Cabinet of the Australian Government (2014).^29^ This estimation was derived from the assumption that an individual in good health would have an average life expectancy of another 40 years.

Sensitivity analysis 3: regarding the CUA, we examined the higher utility scores (0.90, equivalent to population norms^32^ *v*. 0.64) for those who had not self-harmed.

Sensitivity analysis 4: reducing the intervention costs by 20%.

Sensitivity analysis 5: increasing the intervention costs by 20%.

We also conducted two threshold analyses to vary the effect size until the intervention was not cost-saving (ICER = 0) or not cost-effective under the common value-for-money threshold of $50,000 per QALY (ICER = $50 000 per QALY). These threshold analyses were conducted with the assumption of a 50% decay effect regarding intervention effectiveness over the first 5 years.

## Results

### Cost-effectiveness

Table 2 presents results for the base case analysis. The total cost of implementing the Way Back services at a national level was AUD$12.9M (95% uncertainty interval: 11.1M to 14.7M). The modelled intervention subsequently produced AUD$20.4M (95% uncertainty interval: −68.7M to 2.2M) in cost-saving owing to reductions in healthcare costs associated with non-fatal self-harm (AUD$9.9M) and monetary value related to suicide death (AUD$10.5M). The ROI ratio for the intervention was determined to be 1.58 (95% uncertainty interval: −0.17 to 5.33). This meant that for every AUD$1 invested, there would be an estimated return of AUD$1.58. When analysing health outcomes, we found that the theoretical brief aftercare intervention resulted in 222 QALYs gained (95% uncertainty interval: −45 to 563). The mean ICER of the modelled intervention, compared with no intervention, was found to be dominant, with a 95% uncertainty interval ranging from dominant to AUD$189 851 per QALY gained. This indicates that the intervention not only generated positive health benefits but also resulted in net cost-savings.

**Table 2:** Cost-effectiveness summary for the Way Back delivered following self-harm (base case analysis, 50% decay effect up to 5 years)

Output parameter	Value	95% uncertainty interval
Intervention cost (AUD$)	12.9 million	11.1 to 14.7 million
Total cost-saving (AUD$)^a^	−20.4 million	−68.7 to 2.2 million
Healthcare cost-saving (AUD$)^a^	−9.9 million	−18.0 to 3.4 million
Cost-saving associated with death (AUD$)^a^	−10.5 million	−56.7 to 11.8 million
Net costs^a^	−7.5 million	−56.2 to 15.1 million
QALYs	222	45 to 563
ROI ratio	1.58	−0.17 to 5.33
ICER (AUD$ per QALY gained)	Dominant^b^	Dominant^b^ to 189 851
Probability of being cost-effective at $50 000 per QALY gained	87%	–

AUD$, Australian dollars; ICER, incremental cost-effectiveness ratio; QALY, quality-adjusted life year; ROI, return on investment.

a. Negative costs denote cost-savings (if positive costs which denote an expense).

b. A dominant ICER signifies that the intervention is both cost-saving and produces greater health impacts when compared to the comparator.

### Uncertainty and sensitivity analysis

**Fig. 2:**
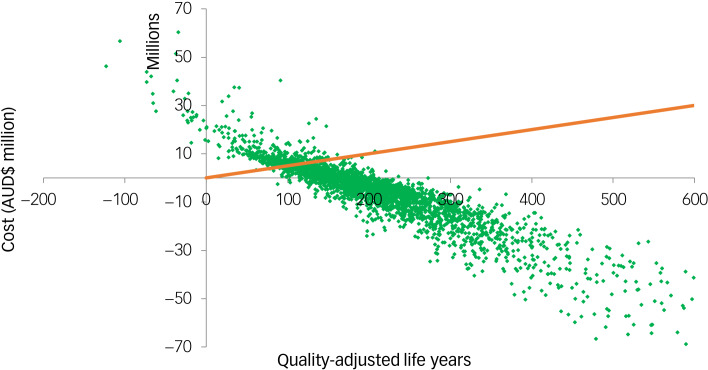
Cost-effectiveness plane of the brief aftercare intervention for suicide prevention: cost–utility analysis.

As shown in Fig. 2, the likelihood of the theoretical brief aftercare intervention being cost-effective at a willingness-to-pay threshold of $50 000 per QALY was 87.0%. Table 3 presents the results of the sensitivity analyses. Overall, the results were robust to changes made across each of the five sensitivity analyses, with ROIs ranging from 1.06 to 6.99. Using the VSL to estimate monetary value on death had the largest impact on the ROI, resulting in a more than fourfold increase in the ROI from 1.58 to 6.99.

**Table 3:** Scenario analysis results

Scenario analyses	50% decay effect ROI ratio, AUD$ saving per AUD$ invested (95% uncertainty interval)	50% decay effect ICER, AUD$ per QALY gained (95% uncertainty interval)	Probability of being cost- effective at $50 000 per QALY
Base case analysis	1.58 (−0.17 to 5.33)	Dominant (dominant to 189 851)	87.0%
Sensitivity analysis 1 – 100% annual decay effect	1.06 (−0.23 to 3.69)	Dominant (dominant to 590 187)	66.3%
Sensitivity analysis 2 – monetary value associated with suicide using value of a statistical life	6.99 (−2.27 to 34.34)	Dominant (dominant to 492 858)	91.7%
Sensitivity analysis 3 – utility score of 0.90 for those who had non-fatal self-harm	1.57 (−0.09 to 5.30)	Dominant (dominant to 69 497)	96.4%
Sensitivity analysis 4 – reducing intervention costs by 20%	1.93 (−0.18 to 6.14)	Dominant (dominant to 167 120)	91.4%
Sensitivity analysis 5 – increasing intervention costs by 20%	1.32 (−0.13 to 4.39)	Dominant (dominant to 232 858)	82.3%

AUD$, Australian dollars; ICER, incremental cost-effectiveness ratio; QALY, quality-adjusted life year.

Threshold analysis indicated that the intervention was no longer cost-saving if it prevented only 20% of people with suicide reattempts (effect size 0.80) at the first year or not cost-effective if it prevented 12% of people with suicide reattempts (effect size 0.88) at the first year and 50% decay of effectiveness within the first 5 years.

## Discussion

The findings of the current study indicate that a theoretical brief aftercare intervention (based on the costs associated with the Way Back Service and the effect size from a meta-analysis of studies not including the Way Back) was likely to be cost-effective within the Australian context, irrespective of whether a ROI analysis or CUA was used. This is the first study to use different economic evaluation frameworks to analyse whether brief aftercare interventions provide good value for money compared with no intervention. The results of this study demonstrated a considerable level of robustness to variations in input parameters and assumptions during the sensitivity analyses. It is worth noting that the monetary value associated with suicide was the parameter with the greatest impact on the cost-effectiveness outcomes. Although there was a high likelihood that the intervention would be considered cost-effective compared with a willingness-to-pay threshold of $50 000 per QALY gained, with two-thirds of uncertainty iterations falling below this threshold, it is important to note that the wide uncertainty intervals around the ICER indicate some probability that the intervention may not be cost-effective when compared to no intervention. Furthermore, threshold analysis indicated that if effect size was reduced by approximately 28%, the intervention would be no longer cost-effective.

The findings of the current model-based evaluation were relatively consistent with previous economic evaluations which support telephone outreach services as a cost-effective intervention for preventing repeated self-harm.^14,15^ Our ROI ratio was slightly lower than that reported in the US study conducted by Richardson et al.^14^ This might have been because our study included services that involve additional intervention components compared with the telephone outreach interventions that were economically evaluated.^14,15^ The Way Back intervention not only included telephone follow-up but also care coordination to assist consumers to stay connected to informal and formal supports. Furthermore, the current study also included training costs associated with the brief aftercare intervention of interest; these were not included in previous economic evaluations. Research to explore the best effective and cost-effective aftercare models for suicide prevention should be performed to support the best funding allocation.

### Study strengths and limitations

The study possesses several notable strengths. First, it employed reasonable and well-founded assumptions based on the most up-to-date literature to construct the economic model, effectively capturing both the benefits and costs associated with the intervention. In addition, the inclusion of two distinct economic evaluation frameworks within a single study context enhanced the robustness of the study's conclusions. The use of both uncertainty analysis and sensitivity analysis in the simulation model further contributed to the study's reliability by enabling us to identify the variables that exerted the greatest influence on the findings.

However, some caution should be exercised in interpreting the results of the study. The evidence of effectiveness of brief aftercare interventions (including telephone contacts, emergency or crisis cards, and postcard or letter contacts) that we used as inputs to the model mostly came from underpowered trials with small sample sizes.^12^ In the meta-analysis by Doupnik et al,^13^ the pooled result for brief aftercare interventions was heavily influenced by one large study^33^ that accounted for a large proportion of the study participants. Nevertheless, even when this study was excluded from the pooled-effects estimate, the combined effect of the interventions consistently indicated a reduction in subsequent suicide attempts.^13^ In addition, the evidence for effectiveness was limited to studies with time horizons of only 1 year. A historical controlled study found that no significant differences were observed between the Way Back intervention and the control group in terms of the proportion of individuals with any readmissions for suicide re-attempts or the number of readmissions during the follow-up period.^18^ Further research on the implementation, acceptability, feasibility and sustainability of brief aftercare interventions is therefore needed so that local, more specific estimates of their short-term and long-term effectiveness can be generated. Furthermore, research to explore which of various different aftercare models are the most effective and cost-effective for suicide prevention should be carried out to support the best funding allocation.

Several limitations may affect the validity of our study's findings. First, the lack of studies with follow-up periods of more than 1 year made it difficult to be certain about the long-term effectiveness of the intervention. Second, the intervention cost was based on the data provided by the Hunter New England Mental Health Services, where only self-poisoning patients were included. This may limit the generalisability of our findings to different healthcare settings, particularly with respect to costs, especially in low- and middle-income countries. Third, the study used a partial societal perspective, which meant that some benefits associated with self-harm prevention were not captured (e.g. impact on carers and family members) (O'Dea & Tucker^34^). This means that the benefit of the intervention may have been underestimated. However, including these costs would make the intervention more favourable with higher ROI.

### Clinical implications

A modelled brief aftercare intervention is likely to be a cost-effective intervention for repeated self-harm prevention for those who have had an index self-harm episode according to both ROI analysis and CUA. Our results indicate that for each AUD$1 invested in 2018, there would be an associated return of AUD$1.58. These findings highlight the potential cost-effectiveness of the intervention. However, further research is necessary to explore the implementation of this intervention in practice and gather more evidence on its effectiveness and long-term outcomes.

## Data Availability

All relevant data are provided in the paper and the supplementary material.

## Supplementary material

For supplementary material accompanying this paper visit http://doi.org/10.1192/bjo.2023.525. click here to view supplementary material
